# Protein pyrrole adducts are associated with elevated glucose indices and clinical features of diabetic diffuse neuropathies

**DOI:** 10.1111/1753-0407.13318

**Published:** 2022-10-04

**Authors:** Xiao Chen, Zhuyi Jiang, Lianjing Zhang, Wei Liu, Xiaohu Ren, Luling Nie, Desheng Wu, Zhiwei Guo, Weimin Liu, Xifei Yang, Yan Wu, Zhen Liang, Peter Spencer, Jianjun Liu

**Affiliations:** ^1^ Key Laboratory of Modern Toxicology of Shenzhen, Shenzhen Medical Key Discipline of Health Toxicology (2020‐2024) Shenzhen Center for Disease Control and Prevention Shenzhen China; ^2^ Department of Endocrinology, Shenzhen People's Hospital (The Second Clinical Medical College Jinan University, The First Affiliated Hospital, Southern University of Science and Technology) Shenzhen China; ^3^ School of Public Health Guangdong Medical University Dongguan People's Republic of China; ^4^ Shenzhen Luohu Hospital for Traditional Chinese Medicine Shenzhen Luohu Hospital Group Shenzhen China; ^5^ Shenzhen Luohu Center for Disease Control and Prevention Shenzhen China; ^6^ Department of Neurology, School of Medicine, and Oregon Institute for Occupational Health Sciences Oregon Health & Science University Portland Oregon USA

**Keywords:** axonopathy, diabetic neuropathy, gamma‐diketone, protein pyrrole adduct, 糖尿病性神经病变, 轴突病, 蛋白质吡咯加合物, γ‐二酮

## Abstract

**Introduction:**

Diabetic neuropathy is the most prevalent complication of diabetes mellitus. Although the precise etiology of this neurological disorder has yet to be defined, elevated blood glucose promotes anerobic glycolysis; this produces excess advanced glycation end‐products, many of which have a pyrrole structure. Here, we test the hypothesis that protein pyrrole adducts are associated with elevated glucose indices and some clinical features of diabetic diffuse neuropathies.

**Method:**

We investigated the levels of plasma pyrrole adducts and adjusted urinary pyrrole adducts in a group of elderly persons (*n* = 516, age 60–79) residing in the District of Luohu, Shenzhen, China between 2017 and 2018. Symptoms of distal symmetric polyneuropathy (DSPN) and resting heart rate, a measure of autonomic nervous system function, were collected from participants (*n* = 258) with elevated glucose indices.

**Result:**

Protein pyrrole adducts showed a strong correlation with glucose indices before and after adjustment for age and estimated glomerular filtration rates. Stratified analysis showed that the medians and interquartile values of pyrrole adducts grew as glucose indices of the subgroups increased. Participants with symptoms of DSPN and sinus tachycardia presented elevated levels of plasma pyrrole adducts.

**Conclusion:**

This study provides a novel link between glucose indices and the etiology of diabetic diffuse neuropathies.

## Graphical abstract

**Highlights**

- The protein pyrrole adducts in both plasma and urine correlated well with glucose indices, namely fasting blood glucose (FBG) and glycate hemoglobin A1c (HbA1c).
- Elevated plasma pyrrole adducts was related to positive distal symmetric polyneuropathy (DSPN) symptoms and a high resting heart rate.
- The findings of protein pyrrole adducts build a new bridge between glucose indices and the etiology of DSPN.

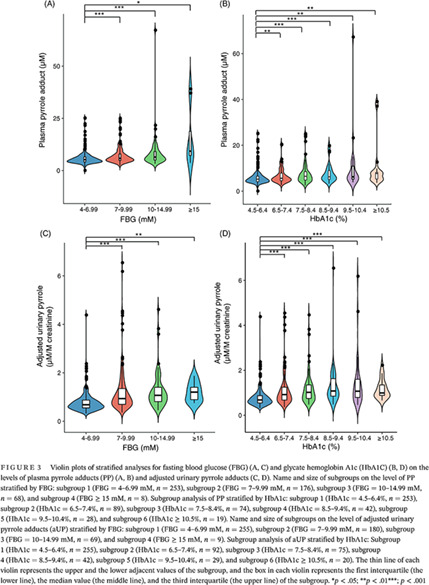

## 摘要

**背景:**

糖尿病神经病变是糖尿病最常见的并发症。虽然这种神经系统疾病的确切病因尚未确定，但血糖升高会促进无氧糖酵解, 这会产生过量的糖基化终产物，其中许多具有吡咯结构。在本文中，我们探究了吡咯蛋白加合物与血糖指数升高和糖尿病弥漫性神经病的一些临床特征的相关性。

**方法:**

我们调查了2017年至2018年间居住在中国深圳罗湖区的一组老年人(n = 516，年龄 60‐79)的血浆吡咯加合物和校正尿吡咯加合物的水平。从血糖指数升高的参与者 (n=258) 中收集远端对称性多发性神经病(DSPN)的症状以及静息心率(衡量自主神经系统功能的指标)。

**结果:**

蛋白质吡咯加合物在校正年龄和估计肾小球滤过率前后与葡萄糖指数有很强的相关性。分层分析表明，吡咯加合物的中位数和四分位数随着亚组葡萄糖指数的增加而增加。有 DSPN 和窦性心动过速症状的参与者血浆吡咯加合物水平升高。

**结论:**

本研究提供了血糖指数与糖尿病弥漫性神经病病因学之间的新关联。

## 1 INTRODUCTION

The US Centers for Disease Control and Prevention states that 11.3% of the US population have diabetes mellitus and 38.0% have prediabetes.^1^ Almost half a billion people are living with diabetes worldwide, and the number is projected to increase by 25% in 2030 and 51% in 2045, when China is expected to have the world's highest number (147 million) of people with the disorder.^2^

Diabetic diffuse neuropathy is a common neurological complication of diabetes mellitus that affects both somatic and autonomic components of the nervous system.^3^ Distal symmetric polyneuropathy (DSPN)—A neurodegenerative disease defined as the presence of symptoms and/or signs of peripheral nerve dysfunction in patients with diabetes after the exclusion of other causes—is the most typical form of diabetic diffuse neuropathies.^4^ Diabetic autonomic neuropathies, particularly cardiovascular autonomic neuropathy (CAN), are also well studied forms of diabetic diffuse neuropathy. Atypical forms of diabetic neuropathy, including diabetic mononeuropathy and amyotrophy (radiculoplexus forms), fall outside the classification of diabetic diffuse neuropathies.^4^

Hyperglycemia dominates the pathogenesis of diabetic diffuse neuropathies. Elevated glucose indices indicate poor metabolic control, which allows diabetic diffuse neuropathies to develop progressively and thereby determine the severity of the neurological disease.^5^, ^6^, ^7^ Elevated blood glucose promotes anerobic glycolysis which in turn produces excess advanced glycation end‐products (AGE), many of which have a pyrrole structure that can react with critical neuroproteins. For example, 3‐hydroxy‐2,5‐hexanodione (3‐HHD), a γ‐diketone analogue generated from the nonenzymatic reaction of the AGE methylglyoxal, forms 3‐hydroxy‐pyrrolated adducts that are detectable in patients with diabetic ketosis.^8^ Because aliphatic and aromatic γ‐diketones are established causes of distal symmetrical axonal neuropathy in humans and laboratory animals,^9^ pyrrole‐protein reactions may be relevant to axonal neuropathies in diabetic states.^10^ Here, we test the hypothesis that both plasma pyrrole adducts (PP) and adjusted urinary pyrrole adducts (aUP) are associated with glucose indices, that is, fasting blood glucose (FBG) and glycate hemoglobin A1c (HbA1c), and these associations are linked with some clinical features of DSPN. Our data are consistent with the hypothesis and set the stage for further clinical studies to examine the relationship between diabetic diffuse neuropathies and protein pyrrole adducts.

## 2 MATERIALS AND METHODS

### 2.1 Study participants

**FIGURE 1:**
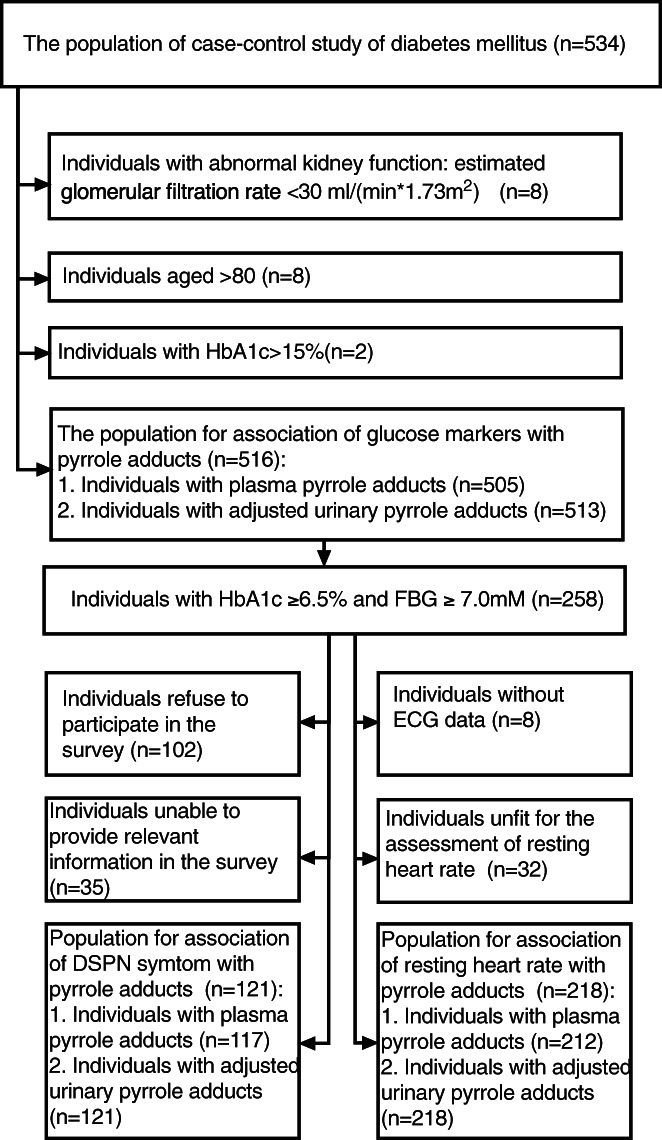
Flow chart of the process of inclusion and exclusion of participants in the study. DSPN, distal symmetric polyneuropathy; ECG, electrocardiography; FBG, fasting blood glucose; HbA1C, glycate hemoglobinA1C

Figure 1 shows that we drew on a population (*n* = 534) of 60–79 year‐old Chinese persons who resided in the Luohu district of Shenzhen City, Guangdong Province, China; these individuals had previously participated in a published case–control study.^11^ Eight persons with abnormal kidney function, eight individuals aged >80 years, and two individuals with extreme glucose levels (HbA1c > 15%) were excluded. Among the 516 remaining individuals, 258 with elevated glucose indices (glycated hemoglobin HbA1c ≥ 6.5% and FBG ≥ 7.0 mM) were shortlisted for assessment of DSPN symptom and resting heart rates. Among these 258 individuals, 156 participated in a phone survey of DSPN symptoms and 121 responded to a questionnaire. The assessment of resting heart rate excluded 40 individuals, eight of whom did not undergo electrocardiography (ECG) and the balance who were unfit for the assessment because (a) they had a clear history of angina pectoris, coronary artery diseases, heart attack, arrhythmia, hepatic failure or cirrhosis, tumors, connective tissue diseases, or psychiatric diseases, or took hormones, beta‐blockers, diuretics, or traditional Chinese medicine; and (b) their electrocardiogram revealed angina pectoris, coronary artery diseases, heart attack, or other arrhythmia.

### 2.2 Physical examination and questionnaire

All study subjects underwent a physical examination and responded to an epidemiological questionnaire between July 2017 to October 2018.^12^ Routine physical examinations included Clinical measurements, imaging, and clinical analysis of blood and urine, for which the following instruments were used: (a) an ultrasonic electronic HNH‐219 height‐and weight scale (Omoron, Kyoto, Japan) to measure body weight with light clothing and height without shoes; (b) a 12‐lead ECG‐1350c electrocardiograph (Nihon Kohden Corporation, Shinjuku, Japan) to obtain the resting ECG; (c) an automatic 7600–010 clinical analyzer (Hitachi Ltd., Tokyo, Japan) to determine the glucose and plasma creatinine level of fasting venous blood; (d) an Hb9210 analyzer (Premier Trinity Biotech, Bray, Ireland) to determine HbA1c levels; (e) an automatic URIT‐500 s urine analyzer (URIT Medical Electronic Group, URIT‐500B, Shenzhen, China) for urinalysis; and (f) a AU5800 Clinical Chemistry Analyzer (Beckman Coulter, Brea, CA) to determine urinary creatinine levels.

The epidemiological instrument that all participants completed on the same day of physical examination included questions relating to sociodemographics (gender, birth date, occupation before retirement), individual and family history of disease, medication history, and lifestyle (active and passive smoking status). Participants with elevated glucose indices (HbA1c ≥ 6.5% and FBG ≥ 7.0 mM) were invited to participate in a short follow‐up phone survey conducted between March and April 2021.The questionnaire was based on the Toronto Clinical Scoring System (TCSS), which emphasizes early sensory symptoms and deficits in DSPN, including the presence or absence of neuropathic pain (burning, stabbing, or shock like); numbness, tingling, and weakness in the feet; the presence or absence of similar upper‐limb symptoms; and the presence or absence of unsteadiness on ambulation (ataxia).^13^ If the participant endorsed any of the six symptoms, if the symptoms were distal and symmetrical in distribution, and had occurred for at least the past 3 years, the subject was regarded as “DSPN symptom positive.” Participants without these criteria were recorded as “DSPN symptom negative.”

Assessment of resting heart rate was based on resting ECG data of participants with elevated glucose indices (HbA1c ≥ 6.5% and FBG ≥ 7.0 mM). Positive subjects were divided into two groups: (a) individuals with a resting heart rate not exceeding 90 bpm were regarded as “sinus bradycardia or normal heart rate,” and (b) those with a heart rate > 90 bpm were reported as “sinus tachycardia.”

### 2.3 Protein pyrrole adducts analysis

4‐Dimethylaminobenzaldehyde (DMBA), 2,5‐dimethylpyrrole (2,5‐DMP) and 14% boron trifluoride methanolic solution (vol./vol.) were purchased from Anpel Laboratory Technologies Inc. (Shanghai, China). Absolute ethanol and hydrochloric acid were obtained from Sinopharm Chemical Reagent Co., Ltd (Shanghai, China). Guanidine chloride was purchased from Tokyo Chemical Industry Ltd (Yubinbango, Aichi, Japan). All reagents were used directly without further purification. Water was distilled and passed through a Merck MilliQ water purification system (Millipore Corporation, Burlington, MA).

Ethylenediaminetetraacetic acid (EDTA)‐anticoagulated fasting venous blood samples and serum were collected for the determination of FBG levels and serum creatinine levels. Plasma samples were separated from the anticoagulated blood by centrifugation and then stored at −80°C prior to determination of PP. The first measurement of optical density (OD_1_) for a mixture of 80 μl plasma and 80 μl of 70% w/v guanidine chloride aqueous solution at 526 nm was recorded with an Infinite M1000 Pro Automatic Microplate Reader (Tecan Ltd, Männedorf, Switzerland). The second measurement of optical density (OD_2_) was recorded at 526 nm after adding 80 μl of Ehrlich's reagent (1% w/v 4‐[dimethylamino] benzaldehyde, DMBA, in 1.5 M hydrochloric acid aqueous solution). The difference in the values of OD_2_ and OD_1_ was calculated and then converted to the concentration of pyrrole adducts by referring to a standard curve for 2,5‐DMP in the range of 0–128 μM.^14^ Urine samples were collected on the morning of physical checking, and these samples were used for the determination of urinary ketone bodies, urobilinogen, and urinary creatinine. Urinary pyrrole adducts were measured in the same way but with the use of a different formulation of Ehrlich's reagent (ie, 3% DMBA in the solution of 40% vol/vol methanolic 14% boron trifluoride and 60% vol/vol ethanol). Results were adjusted based on measured urinary creatinine to obtain measurement of aUP.

### 2.4 Data processing and statistics

Continuous variables that distributed normally were expressed as a mean ± SD. Nonnormally distributed variables were presented as the median and interquartile range and were compared between groups by Wilcoxon rank‐sum test. The Spearman correlation coefficient was used to determine correlations between glucose indices (FBG or HbA1c) and protein pyrrole adducts (PP or aUP); the partial correlation coefficients were calculated with the adjustment of age or duration of diabetes that is applicable for diabetic individuals and estimated glomerular filtration rate (eGFR) were calculated from serum creatinine according to the Chronic Kidney Disease Epidemiology Collaboration creatinine equation.^15^ Statistical analyses were performed using SPSS software (Version 26.0; SPSS, Chicago, IL). A *p* < .05 was considered statistically significant.

## 3 RESULTS

### 3.1 Characteristics of participants

General demographic and medical characteristics of participants, together with their levels of pyrrole adducts, are shown in Table 1. Among all participants, 52.1% (*n* = 269) were female and 47.8% (*n* = 247) were male. The sizes of the age 60–64 subgroup (28.1%), age 65–74 subgroup (34.1%), and the 70–74 subgroup (27.1%) were similar, and the age 75–79 subgroup was much smaller (10.7%). Only a small proportion of participants had weak‐positive to positive results for urinary ketone bodies (3.1%) and urobilinogen (3.1%). A small number of the participants (2.5%) reported prior occupational exposure to organic solvents in the 5‐year period prior to the date of physical examination. The proportions of nonsmokers and current or former smokers were 76.2% and 23.6%, respectively. Based on body mass indices (BMI), the proportions of normal weight (BMI < 23.9), overweight (BMI = 24–27.9), and obesity (BMI > 28) were 48.4%, 38.9%, 12.0%, respectively. For chronic kidney disease (CKD), the proportion of stage‐1 (CKD1), stage‐2 (CKD2), stage‐3a (CKD3a), and stage‐3b (CKD 3b), according to their eGFR, were 16.3%, 71.9%, 9.5%, and 2.3%, respectively. A prior diagnosis of hypertension or coronary heart disease was reported by 60.2% and 6.6% of the participants, respectively.

**TABLE 1:** Descriptive statistics of the study population

Variables	Values
Number of participants (F/M)	269/247
Age^a^ (years)	68.04 ± 4.64
Age (60–64/65–69/70–74/75–79)	145/176/140/55
Fasting blood glucose (mM)^a^	7.48 ± 2.65
HbA1c (%)^a^	7.03 ± 1.55
Urinary ketone bodies (negative/weak positive/positive)	500/15/1
Urobilinogen (negative/weak positive/positive)	500/15/1
Occupational exposure to organic solvent (N/Y)	503/13
Smoking (never/former and current)	393/122
BMI (kg ・ m^−2^)*	24.39 ± 2.92
BMI (<23.9 kg/24–27.9 kg/>28 kg ・ m^−2^)	250/201/62
Estimated glomerular filtration rate (ml/[min × 1.73 m^2^])^a^	74.81 ± 13.60
Stage of kidney functions (eGFR>90/60–89/45–59/30–44 ml/[min × 1.73 m^2^])	84/371/49/12
Hypertension (negative/positive)	205/311
Coronary disease (negative/positive)	478/34
Plasma pyrrole adduct (μM)^b^	5.51(4.43, 7.47)
Adjusted urinary pyrrole adduct (μM/M creatinine)^#^	0.81(0.60, 1.12)

Abbreviations: BMI, body mass indices; eGFR, estimated glomerular filtration rate; F, female, HbA1c, glycate hemoglobin A1c; M, male; N/Y, no/yes.

^a^ Continuous variables that distributed normally are expressed as a mean ± SD.

^b^ Nonnormally distributed variables are presented as the median and interquartile range.

### 3.2 Correlational analysis between glucose indices and pyrrole adducts

**FIGURE 2:**
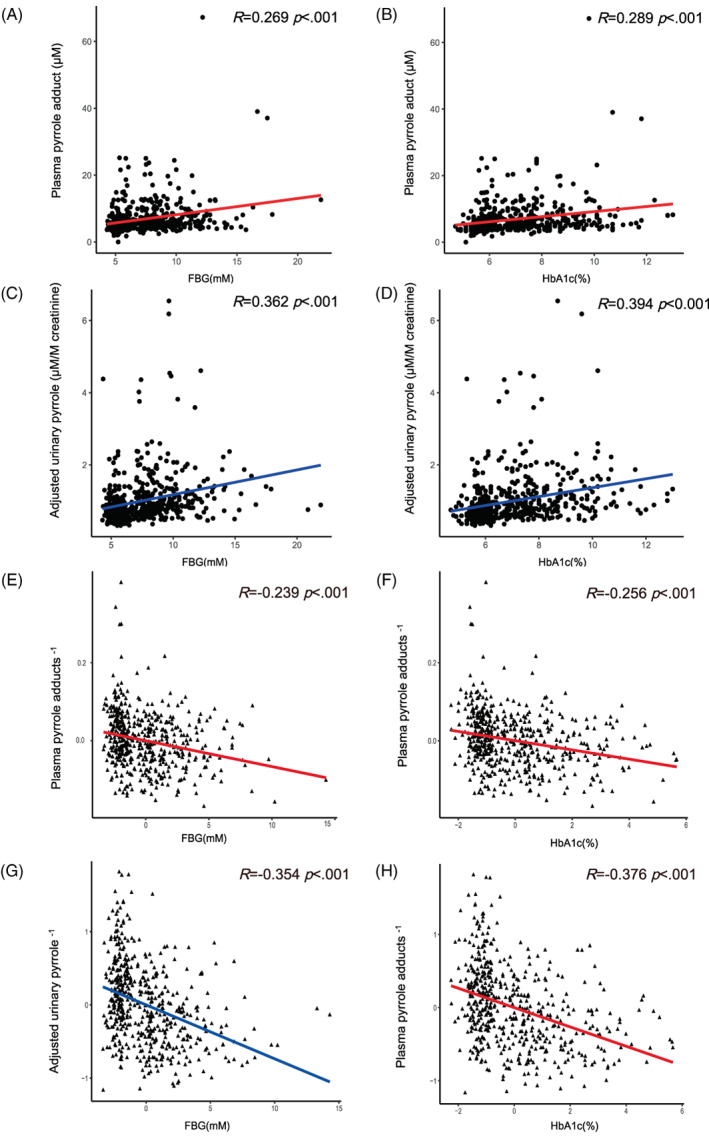
Associations between glucose indices and pyrrole adducts (filled circle): fasting blood glucose (FBG) (A, C) and glycate hemoglobin A1c (HbA1C) (B, D) were significantly associated with plasma pyrrole adducts (PP) (A, B) and adjusted urinary pyrrole adducts (aUP) (C, D). After adjustment for age and estimated glomerular filtration rate (eGFR) (filled triangle), FBG (E, G) and HbA1C (F, H) were significantly associated with reciprocal levels of PP (E, F) and aUP (G, H).

Spearman correlation analyses revealed positive correlations among levels of FBG (Figure 2A, *R* = 0.269) and HbA1c (Figure 2B, *R* = 0.289) and levels of PP (both *p* < .001). A similar positive correlation was observed among levels of FBG (Figure 2C, *R* = 0.362) and HbA1c (Figure 2D, *R* = 0.394) and aUP (both *p* < .001). After adjustment for age and eGFR, partial correlation analyses revealed that both FBG (Figure 2E, *R* = −0.239) and HbA1c (Figure 2F, *R* = −0.256) were negatively associated with reciprocal transformed levels of PP (both *p* < .001). Similar negative associations of FBG (Figure 2G, *R* = −0.354) and HbA1c (Figure 2H, *R* = −0.376) with reciprocal transformed levels of aUP were found (both *p* < .001). Partial correlation analyses that were adjusted for time of diabetes and eGFR. The correlation between reciprocal transformed levels of PP and glucose indices, both FBG and HbA1c, was significant (*p* < .05). The correlation between reciprocal transformed levels of aUP and HbA1c was significant, whereas the correlation between reciprocal transformed levels of aUP and FBG was not significant (*p* = .134).

### 3.3 Subgroup analysis of pyrrole adducts by glucose indices

**FIGURE 3:**
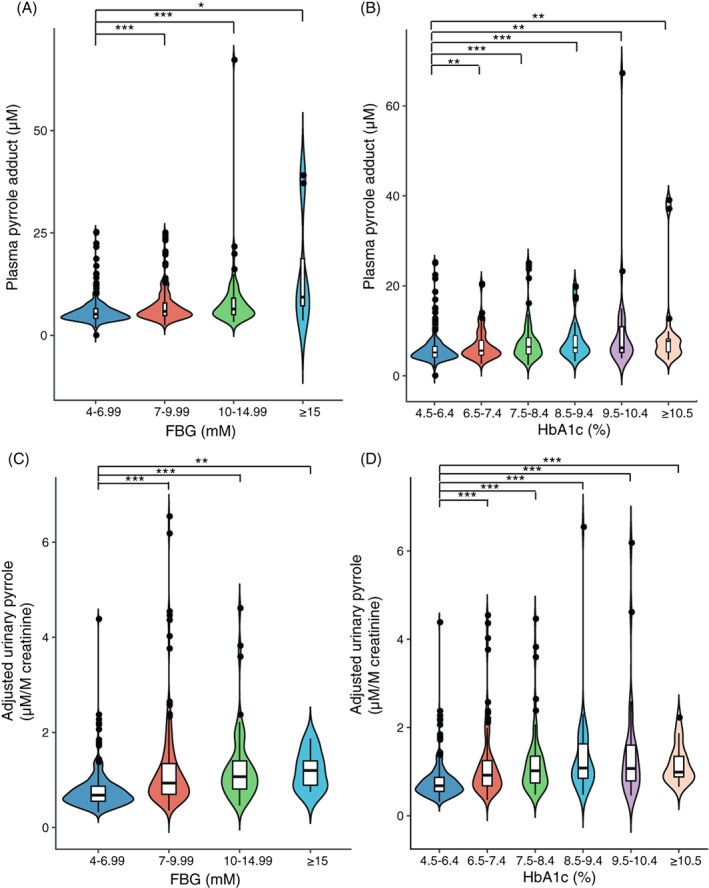
Violin plots of stratified analyses for fasting blood glucose (FBG) (A, C) and glycate hemoglobin A1c (HbA1C) (B, D) on the levels of plasma pyrrole adducts (PP) (A, B) and adjusted urinary pyrrole adducts (C, D). Name and size of subgroups on the level of PP stratified by FBG: subgroup 1 (FBG = 4–6.99 mM, *n* = 253), subgroup 2 (FBG = 7–9.99 mM, *n* = 176), subgroup 3 (FBG = 10–14.99 mM, *n* = 68), and subgroup 4 (FBG ≥ 15 mM, *n* = 8). Subgroup analysis of PP stratified by HbA1c: subgroup 1 (HbA1c = 4.5–6.4%, *n* = 253), subgroup 2 (HbA1c = 6.5–7.4%, *n* = 89), subgroup 3 (HbA1c = 7.5–8.4%, *n* = 74), subgroup 4 (HbA1c = 8.5–9.4%, *n* = 42), subgroup 5 (HbA1c = 9.5–10.4%, *n* = 28), and subgroup 6 (HbA1c ≥ 10.5%, *n* = 19). Name and size of subgroups on the level of adjusted urinary pyrrole adducts (aUP) stratified by FBG: subgroup 1 (FBG = 4–6.99 mM, *n* = 255), subgroup 2 (FBG = 7–9.99 mM, *n* = 180), subgroup 3 (FBG = 10–14.99 mM, *n* = 69), and subgroup 4 (FBG ≥ 15 mM, *n* = 9). Subgroup analysis of aUP stratified by HbA1c: Subgroup 1 (HbA1c = 4.5–6.4%, *n* = 255), subgroup 2 (HbA1c = 6.5–7.4%, *n* = 92), subgroup 3 (HbA1c = 7.5–8.4%, *n* = 75), subgroup 4 (HbA1c = 8.5–9.4%, *n* = 42), subgroup 5 (HbA1c = 9.5–10.4%, *n* = 29), and subgroup 6 (HbA1c ≥ 10.5%, *n* = 20). The thin line of each violin represents the upper and the lower adjacent values of the subgroup, and the box in each violin represents the first interquartile (the lower line), the median value (the middle line), and the third interquartile (the upper line) of the subgroup. *p < .05; **p < .01***; *p* < .001

We conducted subgroup analyses stratified by glucose indices, namely FBG (stratified into four subgroups) and HbA1c (stratified into six subgroups). As shown in Figure 3A, as the FBG level increased from subgroup 1 to 4, the median level of PP rose from 5.20 to 9.31 μM, and the first and third interquartile of that rose from 4.13 to 5.40 μM and from 6.53 to 30.96 μM respectively. The median level of PP of subgroup 1 (5.20 [4.13, 6.53] μM) was significantly lower than that of subgroup 2 (5.86 [4.80, 7.95] μM), subgroup 3 (6.42 [4.98, 9.15] μM), and subgroup 4 (9.31 [5.40, 30.96] μM). As HbA1c (Figure 3B) increased from subgroups 1 to 6, the median level of PP also increased from 5.20 to 7.72 μM, whereas the first interquartile of that rose from 4.13 to 5.19 μM and the third interquartile increased from 6.53 to 8.22 μM. The median PP level of subgroup 1 (5.20 [4.13, 6.53] μM) was significantly lower than that of subgroup 2 (5.59 [4.59, 7.91] μM), subgroup 3 (6.44 [4.82, 8.50] μM), subgroup 4 (6.26 [5.05, 9.19] μM), subgroup 5 (6.22 [5.18, 11.89] μM), and subgroup 6 (7.72 [5.19, 8.22] μM).

Similar trends were observed for levels of aUP stratified by glucose indices. As shown in Figure 3C, as the FBG level increased from subgroup 1 to 4, the median level of aUP continuously increased from 0.68 to 1.20 μM/M creatinine, and the first and third interquartile escalated from 0.68 to 1.20 μM/M creatinine and from 0.88 to 1.55 μM/M creatinine. The median aUP level of subgroup 1 (0.68 [0.55, 0.88] μM/M creatinine) was significantly lower than that of subgroup 2 (0.94 [0.69, 1.36] μM/M creatinine), subgroup 3 (1.07 [0.81, 1.41] μM/M creatinine), and subgroup 4 (1.20 [0.83, 1.55] μM/M creatinine). As HbA1c (Figure 3D) increased from subgroups 1 to 6, the median level of aUP for the first four subgroups (subgroup 1, 2, 3, and 4) maintained an increasing trend, rising from 0.68 to 1.08 μM/M creatinine. The median aUP level of subgroup 1 (0.68 [0.55, 0.88] μM/M creatinine) was significantly lower than that of subgroup 2 (0.92 [0.67, 1.25] μM/M creatinine), subgroup 3 (1.02 [0.74,1.36] μM/M creatinine), subgroup 4 (1.08 [0.84, 1.66] μM/M creatinine), subgroup 5 (1.07 [0.76, 1.61] μM/M creatinine), and subgroup 6 (0.99 [0.86, 1.38] μM/M creatinine).

### 3.4 DSPN symptoms and pyrrole adducts

**FIGURE 4:**
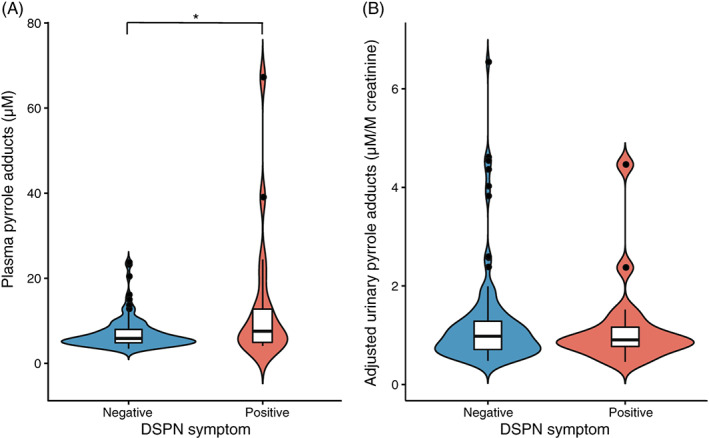
Violin plots of distal symmetric polyneuropathy (DSPN) symptom‐stratified analyses on the levels of plasma pyrrole adducts (PP) (A) and adjusted urinary pyrrole adducts (aUP) (B). The group with positive DSPN symptom, *n* = 16 for PP and *n* = 16 for aUP; the group with negative DSPN symptom, *n* = 101 for PP and *n* = 105 for aUP. The thin line of each violin represents the upper and the lower adjacent values of the subgroup, and the box in each violin represents the first interquartile (the lower line), the median value (the middle line), and the third interquartile (the upper line) of the subgroup. *p < .05

We collected reports of DSPN‐consistent symptoms from participants with elevated glucose indices and analyzed their levels of pyrrole adducts. As shown in Figure 4, participants with DSPN symptoms (8.15 [5.45, 17.99] μM) at the year of physical checking showed significantly higher levels of PPs compared to those of participants without DSPN symptoms (5.86 [4.82, 7.81] μM), whereas aUP levels did not vary between the DSPN‐positive (0.91 [0.74, 1.16] μM/M creatinine) and DSPN‐negative group (0.98 [0.71, 1.32] μM/M creatinine).

### 3.5 Resting heart rate and pyrrole adducts

**FIGURE 5:**
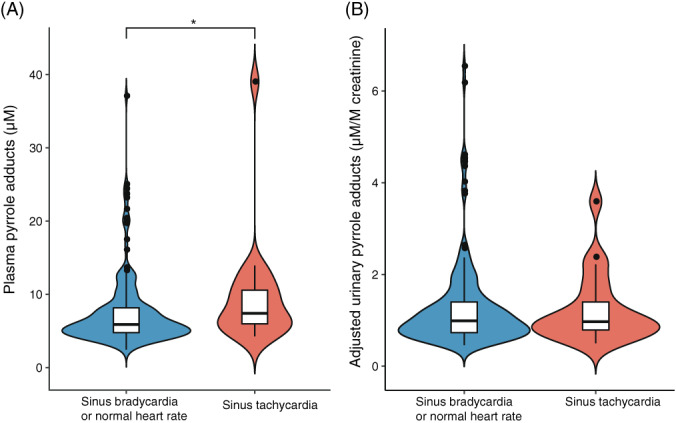
Violin plots of resting heart rate‐stratified analyses on the levels of plasma pyrrole adducts (PP) (A) and adjusted urinary pyrrole adducts (aUP) (B). The thin line of each violin represents the upper and the lower adjacent values of the subgroup, and the box in each violin represents the first interquartile (the lower line), the median value (the middle line), and the third interquartile (the upper line) of the subgroup. The group with sinus tachycardia, *n* = 20 for PP and *n* = 21 for aUP; the group with normal heart rate, *n* = 192 for PP and *n* = 197 for aUP. *p < .05

We analyzed the results of resting heart rate for participants with elevated glucose indices and studied their level of pyrrole adducts. As shown in Figure 5, participants with sinus tachycardia (7.40 [5.82, 10.90] μM) presented significantly higher levels of PP compared to those of participants with a normal heart rate (5.88 [4.74, 8.16] μM), whereas levels of aUP did not vary between the sinus tachycardia group (0.97 [0.74, 1.55] μM/M creatinine) and the normal heart‐rate group (0.99 [0.73, 1.40] μM/M creatinine).

## 4 DISCUSSION

We observed strong positive correlations between protein pyrrole adducts and glucose indices. The four correlation pairs, FBG and PP, HbA1c and PP, FBG and aUP, and HbA1c and aUP (Figure 2), showed consistent *R* (*R* = 0.23–0.28) values, and these correlations were all statistically significant (*p* < .01) before and after adjustment for age and eGFR. Furthermore, the first interquartile, median values, and third interquartile of subgroups rose as glucose indices increased (Figure 3). Despite the complicated pathophysiology of diabetic neuropathy, hyperglycemia is recognized as the driving force, and the control of blood glucose levels is the most common therapeutic intervention. The use of HbA1c in clinical outcomes and settings for diabetic foot peripheral neuropathy has been valorized, whereas HbA1c for biomarker research for diabetic neuropathy requires further attention, especially to identify neurological associations.^16^ In this scenario, plasma protein pyrrole adducts build a new bridge between glucose indices and the etiology of DSPN.

Some of the active metabolites in plasma that form protein pyrrole adducts are associated with hyperglycemia. These include 4‐hydroxy‐2‐nonenal (4‐HNE), a peroxidation product of unsaturated fatty acid that is elevated in the serum of diabetic patients,^17^ the dorsal ganglia root cells of diabetic mice,^18^ the streptozotocin‐induced diabetic rat,^19^ and in zebrafish larvae with impaired glucose homeostasis.^17^ Additionally, serum 4‐HNE levels are increased in patients with type 2 diabetes (who are prone to develop DSPN) and correlate with disease progression.^20^ The 4‐HNE‐derived pyrrole adduct is more thermodynamically stable compared to other protein adducts of 4‐HNE.^21^

Other lipid peroxidation‐derived γ‐ketoaldehydes are also able to form protein pyrrole adducts.^22^ 3‐Deoxyglucosone, an intermediate of glycolysis, forms various pyrrole adducts after reaction with the ϵ‐amino group of lysine. Additionally, pyrraline (e2‐[formyl‐5‐hydroxymethyl‐pyrrol‐1‐yl]‐L‐norleucine), the best known 3‐deoxyglucosone‐derived pyrrole adduct (ie, a type of advanced glycation end product or AGE), is detected in plasma proteins, connective tissue, and the optic nerve head of elderly diabetic patients to a far greater degree than diabetes‐free controls.^23^, ^24^, ^25^ 3‐HHD, a γ‐diketone analog generated from the nonenzymatic reaction of methylglyoxal, forms 3‐hydroxy‐pyrrolated adducts that are detectable in patients with diabetic ketosis.^8^

Post‐translational modification of proteins regulates many biological events like gene expression, protein–protein interaction, and protein processing and degradation. The nucleophilicity of the ϵ‐amino group of lysine makes the lysine residue one of main targets for post‐translational modifications both through enzymatic and nonenzymatic reactions.^26^ Protein pyrrole adducts are formed by Paal–Knoor type reactions between lysine residues and reactive endogenous species, mainly the endogenous γ‐diketone 2,5‐hexanedione (2,5‐HD), lipid peroxidation‐derived keto‐aldehydes, and intermediates of glycolysis. The γ‐diketone analog 2,5‐HD forms 2,5‐DMP adducts with ϵ‐amino groups of lysine residues of proteins and neuroproteins. Experimental animal studies show conclusively that 2,5‐HD triggers a distal symmetrical axonal neuropathy comparable to that found in workers chronically exposed to *n*‐hexane or 2‐hexanone, both of which are metabolized to the γ‐diketone as follows^27^: (a) *n*‐hexane, 2‐hexanol, and 2,‐hexanone undergo phase‐I metabolism to produce 2,5‐HD; (b) 2,5‐HD undergoes Paal–Knoor reaction with ϵ‐amino groups of lysine residues of proteins to form a 2,5‐dimethylpyrrolated protein; (c) the pyrrolated protein undergoes auto‐oxidation that causes protein cross‐linking; (d) cross‐linked proteins involved in the axonal cytoskeleton (neurofilaments) and axonal transport (dynein, kinesin) dysfunction, and energy metabolism is impaired; and (e) abnormal slow axonal transport leads to the accumulation of neurofilaments on the proximal side of distal nodes of Ranvier, localized myelin retraction from the swollen axon, followed by distal axonal degeneration. Toxicokinetic studies have shown PP form dose‐ and time‐dependently in animals treated with *n*‐hexane or 2,5‐HD, and the formation and cross‐linking of protein pyrrole adducts are required steps in the development of γ‐diketone neuropathy.^14^, ^28^, ^29^, ^30^, ^31^ Based on the whole‐body blue chromogenic response to the more potent neurotoxic γ‐diketone (1,2‐diacetylbenzene), it is apparent that protein pyrrole adducts form throughout the body.^32^

Although 2,5‐HD is the ultimate neurotoxic metabolite of *n*‐hexane, it is detectable (mean urinary 2,5‐HD: 0.35–1.47 mg/L, median pyrrole adducts: 0.91–7.4 μM) in populations with no known occupational or environmental exposure to *n*‐hexane or 2‐hexanol.^10^ The origin of endogenous 2,5‐HD is unclear, but it may result from lipid oxidation, which also generates γ‐ketoaldehydes. The commonly used method to detect urinary 2,5‐HD measures both free 2,5‐HD and 4,5‐dihydroxy‐2‐hexanone, ^33^ a reduction product of 3‐HHD.^8^ Moreover, two γ‐diketones (2‐hexanone, 3‐heptanone) with neurotoxic potential, together with 2‐butanone, which potentiates the neurotoxic potential of *n*‐hexane, have been detected in sera of both healthy subjects and those with diabetes mellitus.^34^ Whether the diabetic state results in increased levels of neurotoxic aliphatic γ‐diketones is untested but, if true, this would result in progressive degradation of axonal proteins and contribute to the development of diabetic neuropathy.

Other non‐γ‐diketone‐derived pyrroles may also play role in the pathogenesis of diabetic neuropathy. 4‐HNE is reported to form a pyrrole adduct with apolipoprotein and tau^35^ and to induce oligomerization of α‐synuclein.^36^ 4‐HNE formed pyrrole adducts with mitochondrial and cytoskeleton proteins and induced distal axonal dystrophy and aberrant axonal outgrowth in sensory neurons from diabetic rats.^18^, ^37^ Diversified cross‐linked pyrraline species have also been observed in aging and diabetes.^38^, ^39^, ^40^

The potential for pyrroles to be involved in the genesis of DSPN is consistent with the hypothesis of the present study. We observed correlations between protein pyrrole adducts and some clinical features of DSPN. The diabetic individuals who reported tachycardia and symptoms consistent with DSPN showed elevated PP levels (Figures 4 and 5). Sensory impairments appear to be the most recognizable features of diabetic neuropathy. In one validation study, the correlation between TCSS symptom scores and sural nerve fiber density (FD) was moderate but close to significant (*R* = 0.203, *p* = .0583), which is stronger than that between sensory test scores and FD (*R* = 0.175, *p* = .103).^13^ In the present study, participants with DSPN symptoms had higher PP levels than those without such symptoms. Although tachycardia has been claimed to be the least specific sign of CAN,^41^ a low resting heart rate reflects optimal autonomic function and fitness.^42^ In the present study, the group of participants with a normal heart rate had a significantly lower PP than those in the tachycardia group. Though neither DSPN symptom nor resting heart rate is a sensitive indicator for the diagnosis of DSPN or CAN, the present study still provides some clues linking protein pyrrole adducts and diabetic neuropathy.

The strength of the study is that the biological samples and medical data were ascertained from a large and well‐characterized cohort of elderly individuals. However, an important weakness is the absence of a detailed neurological examination, including sural nerve conduction studies. The DMBA method used here is a semiquantitative method to determine pyrrole adducts and it does not differentiate among types of PP adducts (γ‐diketone‐derived pyrroles, 4‐HNE‐derived pyrroles, and pyrraline all yield positive results with DMBA). Future studies should include detailed clinical (sensory and deep tendon reflex tests) and nerve conduction studies for the diagnosis of DSPN and cardiovascular autonomic reflex tests for the diagnosis of CAN. Limitations in the detection of individual pyrrole adducts may be surmounted with the use of specific antibodies based or mass‐spectrometry based techniques.

## 5 CONCLUSION

The present study provides a novel bridge linking poor glucose control and the etiology of both the somatic and autonomic forms of diabetic neuropathy. The protein pyrrole adducts in both plasma and urine correlated well with glucose indices, namely FBG and HbA1c. Additionally, elevated PPs were related to positive DSPN symptoms and a high resting heart rate.

## AUTHOR CONTRIBUTIONS

Peter Spencer developed the hypothesis. Xiao Chen designed the study and Xiao Chen and Lianjing Zhang performed the analyses of pyrrole markers. Wei Liu, Lianjing Zhang, Xiaohu Ren, and Luling Nie did the survey and managed the human sample. Xiao Chen, Zhuyi Jiang, and Desheng Wu researched the dataset. Wei Liu and Lianjing Zhang did the routine analyses of serum and urine. Xiao Chen and Zhuyi Jiang drafted the manuscript. Weimin Liu, Zhiwei Guo, Xifei Yang, Peter Spencer, and Zhen Liang revised the paper. Yan Wu and Jianjun Liu (guarantor) supervised the study.

## FUNDING INFORMATION

This work was supported by the Guangdong Medical Science and Technology Research Fund (grant number A2022438) Nature Science Foundation of China (grant number 81803209), the Sanming Project of Medicine in Shenzhen (grant number SZSM201611090), and the Shenzhen Key Medical Discipline Construction Fund (SZXK069).

## DISCLOSURE

Not applicable.

## Supporting information


**FIGURE S1** After adjustment for time of diabetes and estimated glomerular filtration rate (eGFR; filled triangle), the association between fasting blood glucose (FBG) and plasma pyrrole adducts (PP) (A) or adjusted urinary pyrrole adducts (aUP) (C) was not statistically significant, whereas the association between glycate hemoglobin A1C (HbA1C) (B, D) and PP (B) and aUP (D) was statistically significant.

## Data Availability

Although the data sets generated and/or analyzed in the current study are not publicly available at this time, they are available to researchers on reasonable request. Specific ideas and proposals for potential collaboration are welcome and should be directed to the corresponding authors, primarily Professor Jianjun Liu (junii8@ 126.com).
