# Supplementary figures and images for: Protein pyrrole adducts are associated with elevated glucose indices and clinical features of diabetic diffuse neuropathies
Source: J Diabetes. 2022 Oct 4;14(10):646–57. doi: 10.1111/1753-0407.13318 (PMC9574754; doi:10.1111/1753-0407.13318)

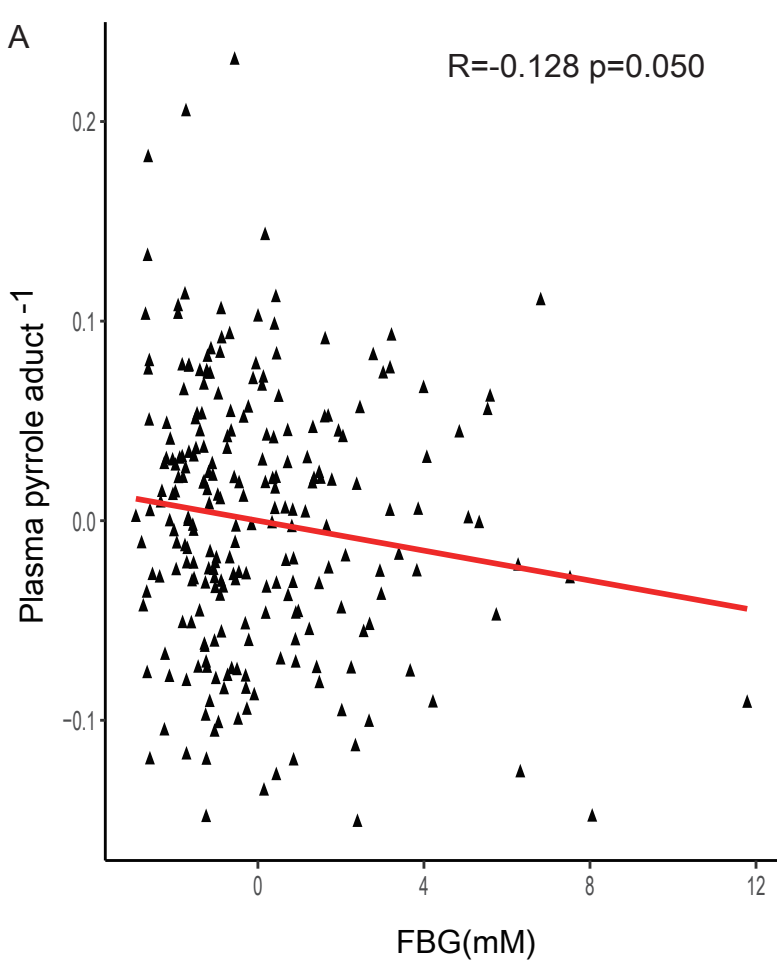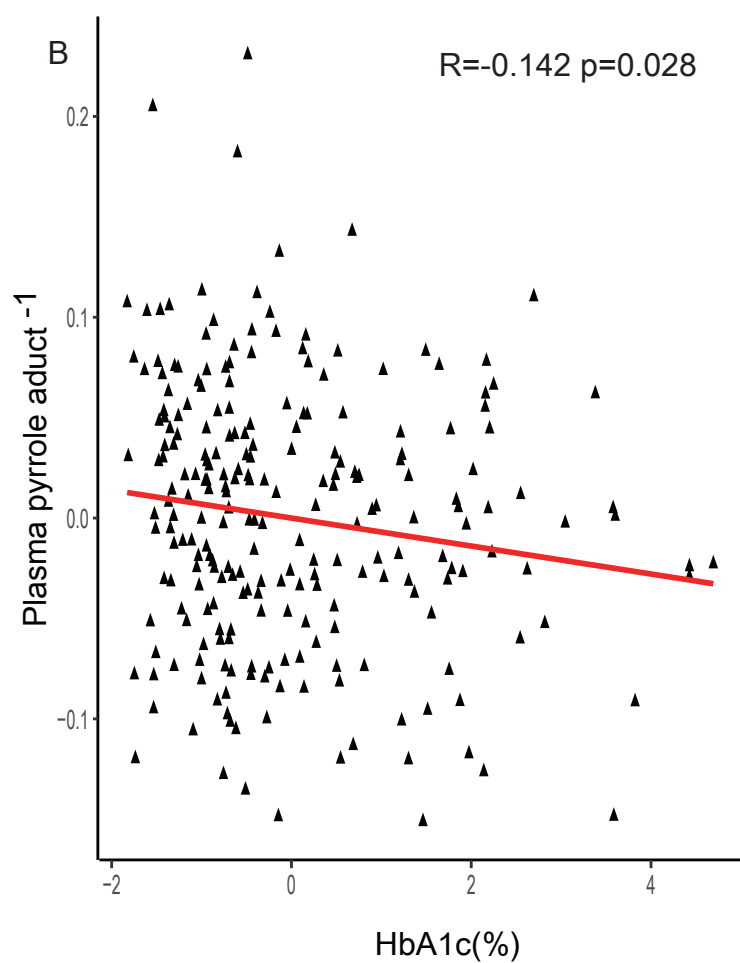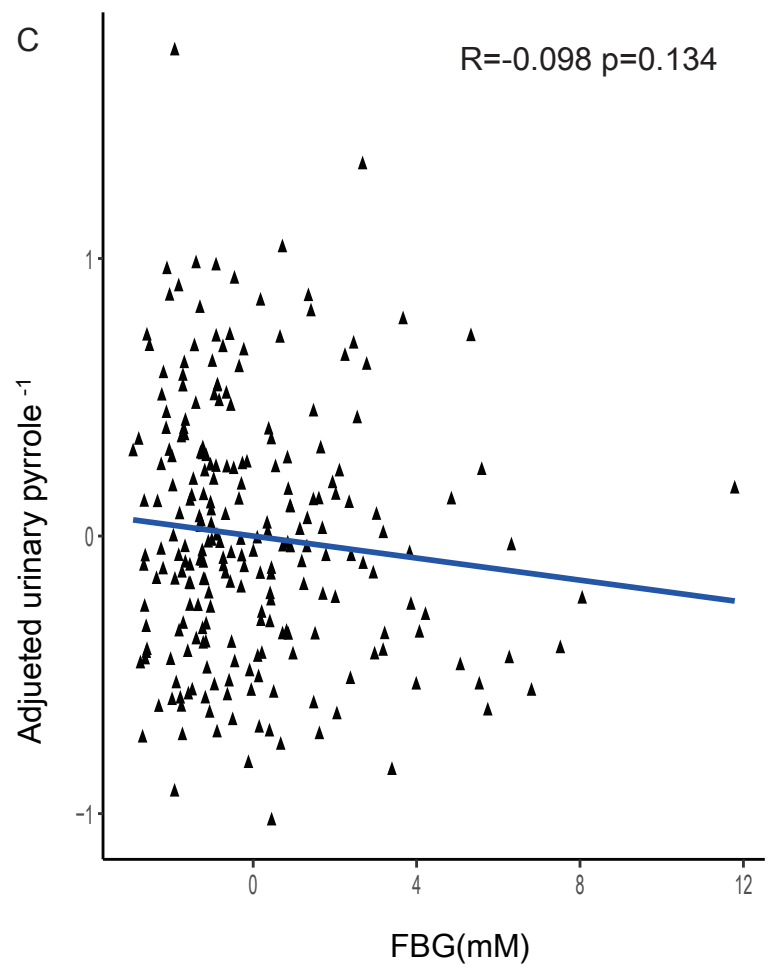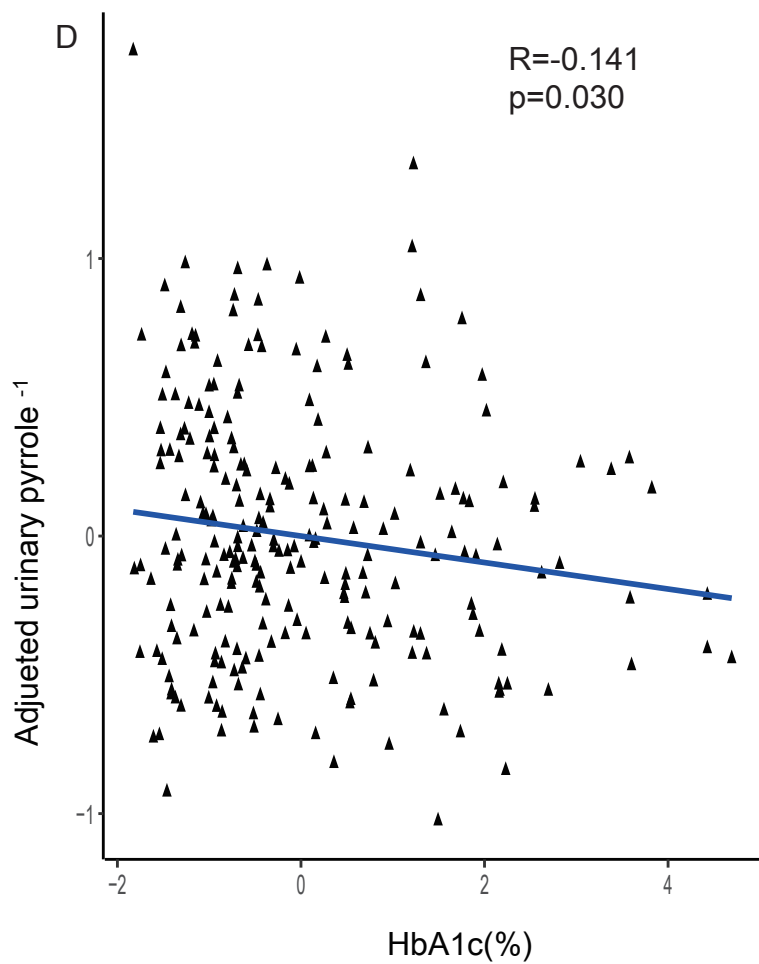

Supplement: Supplementary file 1 — FIGURE S1 After adjustment for time of diabetes and estimated glomerular filtration rate (eGFR; filled triangle), the association between fasting blood glucose (FBG) and plasma pyrrole adducts (PP) (A) or adjusted urinary pyrrole adducts (aUP) (C) was not statistically significant, whereas the association between glycate hemoglobin A1C (HbA1C) (B, D) and PP (B) and aUP (D) was statistically significant. [file JDB-14-646-s001.pdf]
